# *Lactobacillus plantarum* CQPC02 Prevents Obesity in Mice through the PPAR-α Signaling Pathway

**DOI:** 10.3390/biom9090407

**Published:** 2019-08-23

**Authors:** Xin Zhao, Jing Zhang, Sha Yi, Xixi Li, Zemei Guo, Xianrong Zhou, Jianfei Mu, Ruokun Yi

**Affiliations:** 1 Chongqing Collaborative Innovation Center for Functional Food, Chongqing University of Education, Chongqing 400067, China; 2 Chongqing Engineering Research Center of Functional Food, Chongqing University of Education, Chongqing 400067, China; 3 Chongqing Engineering Laboratory for Research and Development of Functional Food, Chongqing University of Education, Chongqing 400067, China; 4 Environment and Quality Inspection College, Chongqing Chemical Industry Vocational College, Chongqing 401228, China

**Keywords:** Sichuan pickle, obese, peroxisome proliferator-activated receptor-alpha (PPAR-α), mice, protein

## Abstract

We determined the lipid-lowering effect of a new strain of the lactic acid bacteria *Lactobacillus plantarum* CQPC02 (LP-CQPC02), from Sichuan pickled cabbages, using an in vivo animal model. A high-fat diet was used to generate obese mice. The effect of LP-CQPC02 was measured using serum parameters and tissues collected from the mice. Obese mice treated with LP-CQPC02 had a lower organ index for liver, epididymal fat, and perirenal fat, and lower levels of aminotransferase (AST), alanine aminotransferase (ALT), triglyceride (TG), and total cholesterol (TC), and low density lipoprotein cholesterol (LDL-C) but higher levels of high density lipoprotein cholesterol (HDL-C) in the serum and liver. LP-CQPC02-treated obese mice also had lower serum levels of the cytokines tumor necrosis factor alpha (TNF-α), interferon gamma (IFN-γ), interleukin 6 (IL-6), and interleukin 1 beta (IL-1β), and higher levels of interleukin 4 (IL-4) and interleukin 10 (IL-10). LP-CQPC02 treatment lessened the obesity-associated pathological changes in the liver and epididymal adipose tissue and reduced adipocyte enlargement. The quantitative PCR (qPCR) and Western blot results showed that LP-CQPC02 treatment up-regulated mRNA and protein expression of lipoprotein lipase (LPL), peroxisome proliferator-activated receptor-alpha (PPAR-α), cholesterol 7 alpha-hydroxylase (CYP7A1), and carnitine palmitoyltransferase 1 (CPT1), but down-regulated peroxisome proliferator-activated receptor-gamma (PPAR-γ) and CCAAT enhancer-binding protein alpha (C/EBP-α) expression in the liver and epididymal adipose tissue. LP-CQPC02 effectively inhibited high-fat diet-induced obesity. The effects of LP-CQPC02 are comparable to the drug l-carnitine but superior to *Lactobacillus delbruechii* subsp. *bulgaricus* (LDSB), which is commonly used in the dairy industry. LP-CQPC02 is a potentially useful, high-quality probiotic strain.

## 1. Introduction

Lactic acid bacteria (LAB) are the main microorganisms used to make traditional fermented foods such as fermented dairy products [1]. LAB may help in the treatment of human gastrointestinal diseases, improve food digestibility and utilization, lower serum cholesterol, and reduce toxins in the body [2,3]. LAB are important in maintaining human digestive balance, manufacturing nutrients, and stimulating tissue development [4]. The special physiological activity of LAB has been widely used in the food industry and in medicine.

Traditional Chinese Sichuan pickled cabbage is naturally fermented cabbage preserved in salt water. Cabbages in a salt solution (5–10%) are fermented by beneficial LAB naturally occurring on the cabbage. The acid production and resulting low pH, together with the high osmotic pressure of the salt solution, inhibit the growth of harmful microorganisms [1]. In the salt solution, LAB utilize sugar and nitrogen-containing substances to grow and produce acidic substances. The metabolism, by the LAB, of chemicals in the brine generates favorable flavors. The amount of LAB present in Sichuan pickled cabbages determines pickle flavor and quality [5]. LAB isolated from fermented foods such as Sichuan pickled cabbages have health benefits including prevention of constipation, colitis, liver damage, and diabetes and they are used as probiotics [6,7]. For example, *Lactobacillus gasseri* and *Lactobacillus coryniformis* have antioxidant activity [8]. *Lactobacillus plantarum* YS4 isolated from naturally fermented foods can prevent constipation in mice [9]. In this study, we isolated the microorganisms in Sichuan pickled cabbages, identified a new strain of *Lactobacillus plantarum* (LP-CQPC02), and determined its physiological activity.

Obesity is a global health concern. Obesity is associated with genetics, endocrine disorders, metabolic abnormalities, and nutritional imbalances [10]. Nutritional excess caused by consumption of convenience foods containing high levels of sugar and fat promotes obesity. Obesity is a risk factor for metabolic diseases such as type 2 diabetes and cardiovascular disease [11]. These metabolic diseases can be prevented by controlling lipid metabolism and preventing obesity [12]. A primary cause of obesity is the imbalance between energy intake and output [13]. High-fat diet induction in C57BL/6J strain mice is a classic animal model for obesity. This model mimics human conditions related to unhealthy eating habits, i.e., ingesting high-fat, high-calorie foods combined with little exercise. Together this results in the accumulation of body fat, elevation of serum total cholesterol (TC) and triglyceride (TG), disorders in carbohydrate, lipid, and protein metabolism, and insulin resistance [14]. Some bioactive food components can activate proliferator-activated alpha receptor (PPAR-α) to stimulate adipocyte differentiation and fatty acid oxidation, and reduce the volume of adipose tissue and size of adipocytes leading to weight loss [15,16]. The PPAR-α pathway is associated with free fatty acid (FFA)-induced lipid accumulation in hepatocytes. Up-regulation of fatty acid oxidation-related genes PPAR-α and carnitine palmitoyltransferase 1 (CPT1) and down-regulation of the SREBP-1 gene can reduce FFA-induced hepatocyte lipid accumulation, regulate lipid metabolism, and thus inhibit weight gain [17]. Studies on probiotic strains or fermented dairy products show that probiotics or metabolites produced during their fermentation are effective in lowering serum cholesterol, visceral fat, and triglyceride levels by altering gut microbiota, gut inflammation, and gut permeability [18]. Studies have shown that in sugar-fat metabolism, *Lactobacillus yoelii* can reduce the secretion of citrate lyase, block the formation of fat and accelerate the oxidation and metabolism of accumulated fat in vivo. In cholesterol-fat metabolism, probiotics can promote the secretion of bile salts hydrolytic enzymes, make bile salts lose their water solubility and become low-water-soluble bile salts, and combine with cholesterol to form precipitation and discharge in vitro, blocking the formation of fat and reducing the content of blood cholesterol [14,17].

In this study, we administered LP-CQPC02, isolated from Sichuan Pickled cabbages, to mice fed a high-fat diet (model for diet-induced obesity). The lipid-lowering effect of LP-CQPC02 was investigated by measuring appropriate outcome parameters in the serum and tissues of these mice. We also determined the involvement of the PPAR-α pathway in the action of LP-CQPC02.

## 2. Materials and Methods

### 2.1. Experimental Strain

*Lactobacillus plantarum* CQPC02 was isolated from Sichuan pickle fermentation water in Chongqing, China. LP-CQPC02 was identified using BLAST (Basic Local Alignment Search Tool) in NCBI; it had 99% homology with NC_004567.2 strain in NCBI and was identified as *L. plantarum*. LP-CQPC02 has been preserved in China General Microbiological Culture Collection Center (Beijing, China). *Lactobacillus delbruechii* subsp. *bulgaricus* (China Center for Type Culture Collection, Wuhan, Hubei, China) was used as a positive control.

### 2.2. Resistance of Lactobacillus to 0.3% Bile Salt

In the de Man, Rogosa and Sharpe (MRS)-THIO medium (containing 0.2% sodium thiol acetate MRS broth), the pig bile salt was added at a concentration of 0.3% and 121 °C for sterilization at 15 min. The activated 5 mL strain was inoculated with 2% (*v/v*) inoculation volume into MRS-THIO medium without bile salt (0%) and MRS-THIO medium containing 0.3% bile salts. MRS-THIO medium was used as a control. Optical density (OD)_600_ nm values of the above-mentioned media at different concentrations were measured after incubation at 37 °C for 24 h. The tolerance of strains to bile salt was calculated according as bile salt tolerance (%) = (OD_600_ nm of 0.3% bile salt medium − OD_600_ nm of blank medium)/(OD_600_ nm of 0.0% bile salt medium − OD_600_ nm of blank medium) × 100.

### 2.3. Tolerance Test of Artificial Gastric Juice

Artificial gastric juice consisted of 0.2% NaCl and 0.35% pepsinase. NaCl and pepsinase required for the test were weighed according to the corresponding mass-volume ratio. The pH of the prepared artificial gastric juice was adjusted to 3.0 with 1 mol/L HCl, and then filtered with a 0.22 μm membrane to remove bacteria. The protocol was as follows: absorbing 5 mL cultured bacteria from a super-clean workbench in a 10 mL sterile centrifugal tube, centrifuging for 10 min at 3000 r/min, discarding the upper culture medium and collecting bacteria, adding 5 mL sterile saline to mix to make bacterial suspension, then taking 1 mL bacterial suspension and 9 mL pH 3.0 artificial gastric juice to mix, at this time, take 1 mL. The mixture was treated with artificial gastric juice for 0 h. The remaining 9 mL mixture was cultured in a constant temperature water bath shaking bed (37 °C, 150 r/min) for 3 h. The samples of 0 h and 3 h were diluted by a 10 times gradient, and the suitable gradient was selected to determine the number of viable bacteria by the plate coating method. The samples were cultured on MRS solid medium at 37 °C for 48 h; the survival rate (%) was calculated according to formula: survival rate (%) = 3 h viable count (CFU/mL)/0 h viable count (CFU/mL) × 100.

### 2.4. Inducing Obesity in Mice

Fifty male and female (1:1), 6-week-old specific pathogen-free C57BL/6J mice (Chongqing Medical University, Chongqing, China) were acclimatized for seven days before being divided into five treatment groups (10 mice/treatment): normal, model, LP-CQPC02, l-carnitine, and *Lactobacillus delbruechii* subsp. *bulgaricus* (LDSB). Mice in the normal group were fed a low-fat diet (Table 1), and those in the remaining groups were fed a high-fat diet for 8 weeks. During the 8 weeks of feeding, mice in the LP-CQPC02 group and LDSB group received a daily intragastrical gavage of LP-CQPC02 and LDSB (both at 1.0 × 10^9^ CFU/kg body weight), respectively, and mice in the l-carnitine group received a daily intragastrical gavage of l-carnitine (200 mg/kg body weight). Body weight was recorded weekly. At the end of the study, mice were not fed for 24 h prior to euthanization. Blood was collected by heart puncture, and the liver, epididymal fat, and perirenal fat were collected and weighed for organ index calculation and analysis. The weights of liver tissue, epididymis adipose tissue and perirenal adipose tissue were measured. The organ index was calculated by the following formula: organ tissue index = organ weight (g)/body weight (g) ×100. The experimental mice blood samples were drawn from orbit. The protocol for these experiments was approved by the Ethics Committee of Chongqing Collaborative Innovation Center for Functional Food (201804004B), Chongqing, China.

### 2.5. Determination of ALT, AST, HDL-C, LDL-C, TC and TG Levels in Serum and Liver of Mice

The obtained plasma was centrifuged at 4000 rpm and 4 °C for 10 min and then the upper serum was taken. The levels of alanine aminotransferase (ALT), aminotransferase (AST), HDL-C, LDL-C, total cholesterol (TC), triglyceride (TG), low density lipoprotein cholesterol (LDL-C) and high density lipoprotein cholesterol (HDL-C) in the serum of mice were determined according to the kit instructions (Nanjing Jiancheng Bioengineering Institute, Nanjing, Jiangsu, China). The liver of mice was homogenized into 10% homogenate (1 g) and centrifuged at 4000 rpm and 4 °C for 10 min. The supernatant was taken, and the liver tissue index was determined according to their respective kit instructions (Nanjing Jiancheng Bioengineering Institute).

### 2.6. Determination of TNF-α, IFN-γ, IL-1β, IL-4, IL-6 and IL-10 Cytokine Levels in Serum of Mice

The serum cytokine levels of tumor necrosis factor (TNF)-α, IFN-γ, interleukin (IL)-1β, IL-4, IL-6 and IL-10 in mice were determined according to their respective kit instructions (Abcam, Cambridge, MA, USA).

### 2.7. Pathological Observation

Mouse liver and epididymal adipose tissues were put in 10% formalin for 48 h. The formalin-fixed tissues were then dehydrated, paraffin-embedded, and sectioned for H&E (hematoxylin-eosin staining) staining. The tissue histology was examined under an optical microscope (BX43, Olympus, Tokyo, Japan).

### 2.8. Quantitative PCR Assay

Liver and epididymal adipose tissues were homogenized in a sample homogenizer. Total RNA in tissues was extracted with TRIzol (Thermo Fisher Scientific, Waltham, MA, USA) using the manufacturer’s protocol, and the extracted total RNA was diluted to 1 μg/μL. One microliter of total RNA was used for reverse transcription to generate cDNA according to the manufacturer instructions (Thermo Fisher Scientific). PCR was conducted by mixing 1 μL of cDNA template with 10 μL of the SYBR Green PCR Master Mix (Thermo Fisher Scientific), 1 μL of primers (1 μg/μL) (Table 2), and 7 μL of sterile distilled water. PCR was conducted under the following conditions: 95 °C for 60 s; 40 cycles of 95 °C for 15 s, 55 °C for 30 s, and 72 °C for 35 s; 95 °C for 30 s; 55 °C for 35 s. A stable single GAPDH expression was selected as an internal reference gene according to MIQE guidelines. The 2^−ΔΔCt^ method was used to calculate the relative expression of genes [19].

### 2.9. Western Blot

Liver and epididymal adipose tissues (100 mg) in 1 mL radio immunoprecipitation assay (RIPA) solution were homogenized at 12,000 rpm, 4 °C for 5 min, followed by centrifugation at 12,000 rpm, 4 °C for 15 min. The intermediate layer that contained protein was collected, and protein concentrations were measured using a BCA protein quantification kit (Thermo Fisher Scientific) according to the instructions. Samples were diluted to 50 μg/mL and then mixed with the sample buffer at a ratio of 4:1. Samples were heated at 100 °C for 5 min and then put on ice for 5 min. They were then loaded on a sodium dodecyl sulfate polyacrylamide gel electrophoresis (SDS-PAGE) gel for separation. A pre-stained protein ladder was loaded as markers for the molecular weight of proteins. SDS-PAGE gel was subjected to 50 min of vertical gel electrophoresis, followed by transfer to the methanol-activated PVDF membrane (Thermo Fisher Scientific). After transfer, PVDF membrane was blocked by a 1× tris buffered saline tween (TBST) solution containing 5% skim milk for 1 h. After the PVDF membrane was washed with 1× TBST, the primary antibody (Thermo Fisher Scientific) was added and incubation was conducted at 25 °C for 2 h. Polyvinylidene fluoride (PVDF) membrane was then washed 5 times with 1× TBST before the secondary antibody was added and incubated at 25 °C for 1 h. MagicMark XP Western (Thermo Fisher Scientific) was used as protein molecular weight marker. Finally, the Supersignal West Pico PLUS agent was added to the PVDF membrane and fluorescence signals were detected in the iBright FL1000 (Thermo Fisher Scientific) as previously reported [20].

### 2.10. Statistical Analysis

The serum and tissue determination experiments of each mouse were conducted three times in parallel, and then the mean values were taken. Then, the data were analyzed by using the Statistical Package for the Social Science statistical software (SAS 9.1, SAS Institute Inc., Cary, NC, USA). One-way ANOVA was used to analyze whether there were significant differences among groups of data at the level of *p* < 0.05.

## 3. Results

### 3.1. Resistance of Lactic Acid Bacteria to Artificial Gastric Juice and Bile Salt

Table 3 shows that the survival rate of LP-CQPC02 in artificial gastric juice at pH 3.0 was more than 90%, which was much higher than that of LDSB. The survival rate of LP-CQPC02 in 0.3% bile salt was close to 20%, which was more than twice that of LDSB. This shows that LP-CQPC02 has strong tolerance to bile salt and artificial gastric acid.

### 3.2. Body Weight of Mice

**Figure 1:**
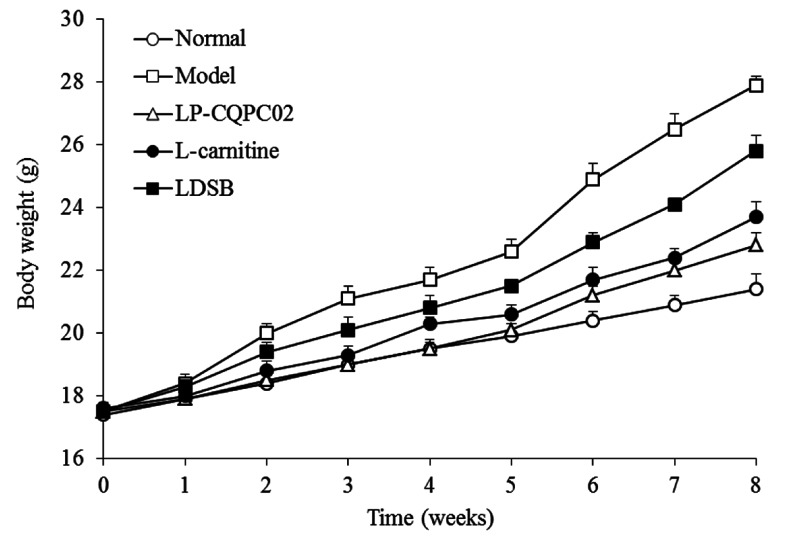
Body weight of mice during the experiment. LDSB: Mice treated with 1.0 × 10^9^ CFU/kg of *Lactobacillus delbruechii* subsp. *bulgaricus*; LP-CQPC02: Mice treated with 1.0 × 10^9^ CFU/kg of *Lactobacillus plantarum* CQPC02; l-carnitine: Mice treated with 200 mg/kg of l-carnitine.

Figure 1 shows that the normal group mice had a slight weight gain while mice in the model group had a larger weight gain. Mice in l-carnitine, LP-CQPC02, and LDSB groups had lower weight gains compared to the model group. Weight gain in the LP-CQPC02 group was lower than that in the l-carnitine or LDSB group. LP-CQPC02 treatment prevented high-fat diet-induced weight gain and suggests that it may have had an anti-obesity effect.

### 3.3. Organ Indices of Mice

Table 4 shows that the organ index for the liver, epididymal fat, and perirenal fat in the model group was significantly higher (*p* < 0.05) than in the control group. This indicates that the high-fat diet was associated with liver enlargement and increased volume of epididymal and perirenal fat. The high-fat diet changes were significantly smaller in the l-carnitine, LP-CQPC02, and LDSB groups compared to the model group (*p* < 0.05). The LP-CQPC02 and l-carnitine group values were similar to the values in the normal group. This result suggests that the high-fat diet did not induce increase in organ index or excessive fat accumulation in organs under LP-CQPC02 treatment.

### 3.4. Levels of ALT, AST, HDL-C, LDL-C, TC and TG in Serum and Liver of Mice

The serum levels of ALT, AST, TC, TG, and LDL-C were lowest in the normal group and highest in the model group, while the reverse was true for the HDL-C levels (Table 5 and Table 6). The levels of ALT, AST, TC, TG, and LDL-C were significantly lower and the HDL-C level was significantly higher in LDSB, LP-CQPC02, and l-carnitine groups compared to the model group (*p* < 0.05). These effects were greatest in the LP-CQPC02 and l-carnitine groups and their values for these measures were similar to values in the normal group. These results indicate that LP-CQPC02 was effective in preventing high-fat diet-induced obesity. Its effect was comparable to l-carnitine but greater than LDSB.

### 3.5. Cytokine Levels of TNF-α, IFN-γ, IL-1β, IL-4, IL-6 and IL-10 in Serum of Mice

The model group had significantly higher serum levels of the cytokines TNF-α, IFN-γ, IL-6, and IL-1 β but significantly lower levels of IL-4 and IL-10 compared to all the other groups (*p* < 0.05, Table 7). All these changes were reduced by treatment with LDSB, LP-CQPC02, or l-carnitine, with LP-CQPC02 being the most effective.

### 3.6. Hematoxylin-Eosin Pathological Observation

**Figure 2:**
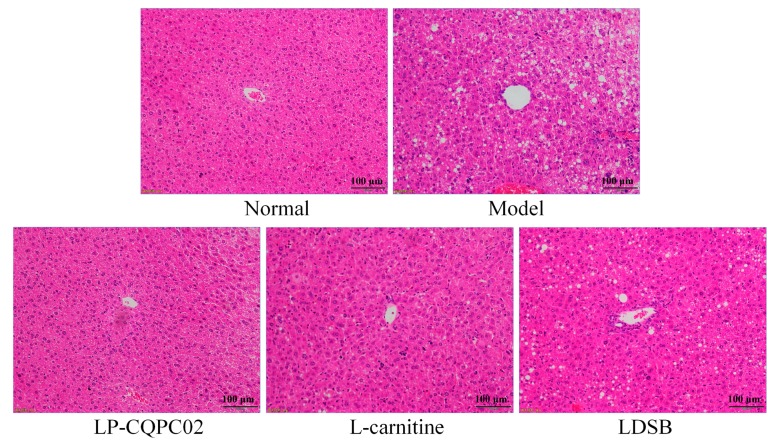
Hematoxylin-eosin (H&E) pathological observation of hepatic tissue in mice. Magnification 100×. LDSB: Mice treated with 1.0 × 10^9^ CFU/kg of *Lactobacillus delbruechii* subsp. *bulgaricus*; LP-CQPC02: Mice treated with 1.0 × 10^9^ CFU/kg of *Lactobacillus plantarum* CQPC02; l-carnitine: Mice treated with 200 mg/kg of l-carnitine.

H&E staining showed that hepatocytes of mice in the normal group had no abnormalities such as steatosis. The liver had an intact structure, normal lobular architecture, clear cell boundaries, and centrally located nuclei (Figure 2). In contrast, liver tissue from the mice in the model group showed vesicular steatosis, increased fat content, large numbers of fat vacuoles around the blood vessels, swollen cells, and reduced cell wall integrity. These pathological changes were attenuated by LP-CQPC02 treatment to levels similar to the normal group.

**Figure 3:**
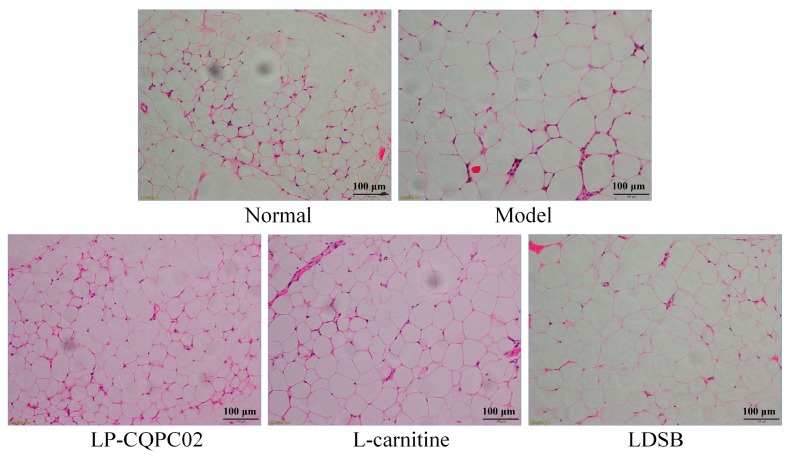
H&E pathological observation of epididymal tissue in mice. Magnification 100×. LDSB: Mice treated with 1.0 × 10^9^ CFU/kg of *Lactobacillus delbruechii* subsp. *bulgaricus*; LP-CQPC02: Mice treated with 1.0 × 10^9^ CFU/kg of *Lactobacillus plantarum* CQPC02; l-carnitine: Mice treated with 200 mg/kg of l-carnitine.

Histology of epididymal adipose tissue showed that adipocytes were smaller and evenly arranged in the normal group while in the model group, adipocytes were larger and had thinner cell membranes (Figure 3). Adipose tissue in the LP-CQPC02 and l-carnitine groups was denser than that in the model group, and cell size was similar to the normal group; the effects of LP-CQPC02 or l-carnitine treatment were greater compared to LDSB treatment. These results indicate that LP-CQPC02 can reduce high-fat diet-induced adipocyte hypertrophy with an efficacy similar to l-carnitine.

### 3.7. RNA and Protein Expression in Liver and Epididymis Tissue of Mouse

**Figure 4:**
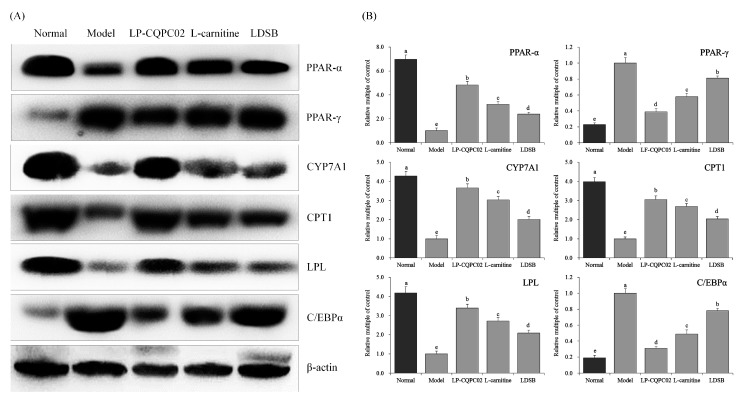
The peroxisome proliferator-activated receptor-alpha (PPAR-α), peroxisome proliferator-activated receptor-gamma (PPAR-γ), cholesterol 7 alpha-hydroxylase (CYP7A1), carnitine palmitoyltransferase 1, lipoprotein lipase (LPL) and CCAAT enhancer-binding protein alpha (C/EBP-α) mRNA (**A**) and protein (**B**) expression in hepatic tissue of mice. ^a–e^ Mean values with different letters above the bar are significantly different (*p* < 0.05) according to Duncan’s multiple-range test. LDSB: Mice treated with 1.0 × 10^9^ CFU/kg of *Lactobacillus delbruechii* subsp. *bulgaricus*; LP-CQPC02: Mice treated with 1.0 × 10^9^ CFU/kg of *Lactobacillus plantarum* CQPC02; l-carnitine: Mice treated with 200 mg/kg of l-carnitine.

**Figure 5:**
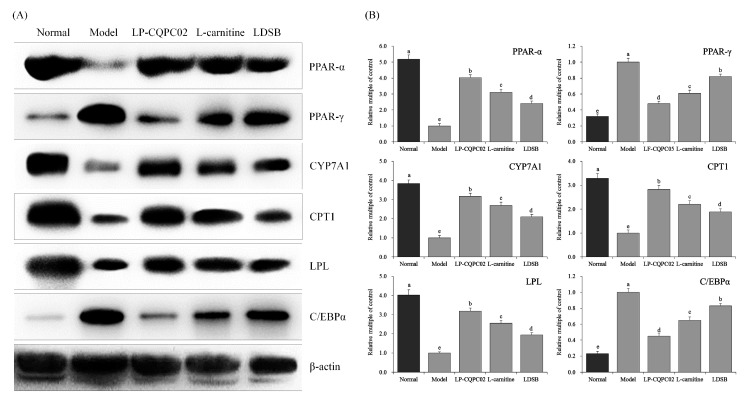
The PPAR-α, PPAR-γ, CYP7A1, CPT1, LPL and C/EBPα mRNA (**A**) and protein (**B**) expression in epididymis tissue of mice. ^a–e^ Mean values with different letters above the bar are significantly different (*p* < 0.05) according to Duncan’s multiple-range test. LDSB: Mice treated with 1.0 × 10^9^ CFU/kg of *Lactobacillus delbruechii* subsp. *bulgaricus*; LP-CQPC02: Mice treated with 1.0 × 10^9^ CFU/kg of *Lactobacillus plantarum* CQPC02; l-carnitine: Mice treated with 200 mg/kg of l-carnitine.

Among the different treatment groups, mRNA and protein expression of LPL, PPAR-α, CYP7A1, and CPT1 were lowest while PPAR-γ and C/EBPα expression were highest in the model group. For all of these measures, the patterns were reversed in the normal group (Figure 4 and Figure 5). All the high-fat diet-induced changes were significantly attenuated in LDSB, LP-CQPC02, and l-carnitine groups (all *p* < 0.05). LP-CQPC02 and l-carnitine were superior to the LDSB treatment. LP-CQPC02 could inhibit the negative impact of high-fat diet-induced obesity on mRNA and protein expression of some marker molecules. Its inhibition efficacy was similar to l-carnitine and superior to the commonly used commercial strain LDSB.

## 4. Discussion

LDSB is the most common lactic acid bacteria used in yoghurt fermentation and probiotics. In order to compare the effects of LP-CQPC02 in this study, we chose LDSB for comparison. Under laboratory conditions, by observing the survival rate of lactic acid bacteria in artificial gastric juice and bile salt, the resistance of lactic acid bacteria can be judged, and the possibility of colonization of lactic acid bacteria into the intestine can be preliminarily judged [3]. In this study, LP-CQPC02 had better anti-bile salt and anti-gastric acid ability than commercial strain LDSB, and could better enter the intestinal tract of mice for colonization.

In weight loss experiments, body weight is a primary index and changes in the organ index are also used to assess fat accumulation and lipid metabolism. When mice become obese, there is an increase in the percent of total white fat in their total body weight. Because epididymal fat is typical white fat, determining white fat weight and its percentage of total body weight are indices of obesity [21]. A high-fat diet can induce a stress response with hepatic lipid accumulation leading to hepatomegaly and reduced liver function. Under normal conditions, lipid synthesis and export to hepatocytes are balanced so that there is no lipid accumulation in the liver and no significant formation of lipid droplets in the cytoplasm of hepatocytes. However, during obesity, lipid will form droplets of varying sizes leading to loss of the normal structure and function of liver cells. Because the organ index can reflect the changes in organ structure and function, it can be used to assess efficacy of intervention in affecting weight loss in animal models. Our results showed that the high-fat diet caused a significant increase in the organ index in mice. This increase was effectively prevented by LP-CQPC02 or l-carnitine treatment as indicated by a reduced increase in the organ index and reduced weight gain.

The liver is critical for detoxification and lipid metabolism. Serum levels of ALT, AST, and ALP are important diagnostic indicators of liver function. These enzyme indicators reflect the level of damage to liver function [22]. ALT and AST, enzymes mainly present in hepatocytes, are released into circulation during hepatocyte necrosis and this results in increased serum levels of these enzymes. These increased enzyme levels parallel the extent of cell damage and they are often used as indicators of liver function [23]. An abnormal blood lipid profile (including levels of TG, TC, HDL-C, and LDL-C) is a common indicator of metabolic syndrome, and it reflects systemic lipid metabolism [24]. An excessive increase in body fat is the main cause of obesity. About 30–50% of individuals with high blood lipids have a fatty liver, a reversible condition that can return to normal after early diagnosis and treatment [25]. We found that consumption of a high-fat, high-calorie diet caused obesity and significant elevation of blood ALT, AST, ALP, TG, TC, and LDL-C in mice. These changes were accompanied by severe vacuolation and lipid deposition in the liver. Administration of LP-CQPC02 to mice fed high-fat diet reduced ALT, AST, ALP, TG, TC, and LDL-C levels and lipid accumulation in the liver and also increased HDL-C levels.

Increased visceral fat, organ damage, and inflammation are observed in the obese state. IL-6, IL-1β, TNF-α, and IFN-γ as proinflammatory cytokines can promote inflammation and cause tissue damage [26]. Among them, IL-1β can promote the action of adhesion molecules [27]. In the initial stage of inflammation, TNF-α induces production of cytokines, and it also works with these cytokines in regulating immune responses [28]. IL-4 and IL-10 are anti-inflammatory factors that can inhibit inflammation and help maintain homeostasis; by doing so they are involved in immune regulation and organ protection [29]. In the current study we found that LP-CQPC02 treatment significantly inhibited obesity-associated inflammation and tissue damages in mice fed a high-fat diet.

**Figure 6:**
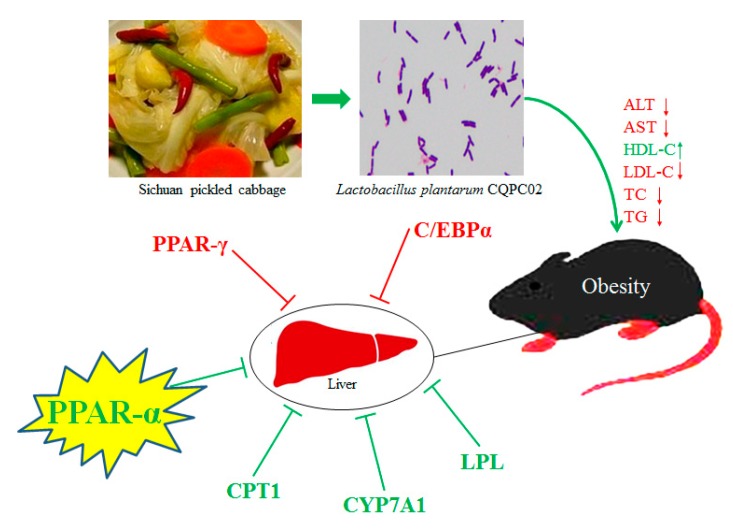
Action mechanism of *Lactobacillus plantarum* CQPC02.

PPAR is a member of the nuclear transcription factor superfamily that regulates expression of target genes. PPAR can be activated by ligands such as fatty acid-like compounds and it plays an important role in cell signaling. PPAR participates in a variety of metabolic processes, such as respiratory chain of oxidation and fatty acid β oxidation. It is also involved in physiological and pathological processes in the development of several diseases as well as obesity [30]. PPAR includes three subtypes (α, β, γ). PPAR-γ is expressed primarily in adipose tissue, and it regulates adipocyte formation and differentiation. PPAR-γ not only regulates expression of the genes related to lipid metabolism but is also a key regulator for adipocyte gene expression and insulin signaling in cells [31]. C/EBP-α is a transcription factor involved in regulating adipocyte differentiation. C/EBP-α and PPAR-γ are reported to work together in a synergistic manner. Activation of PPAR-γ can induce C/EBP-α gene expression, and expression of these two transcription factors is positively correlated [32]. CPT1, a rate-limiting enzyme in fatty acid oxidation, can change in response to obesity. CPT1 has its C-terminal positioned toward the outer membrane of mitochondria, and its N-terminal toward the cytoplasm. CPT1 regulates the rate of fatty acid synthesis by converting acyl coenzyme A to acylcarnitines [33]. PPAR-α is an upstream transcription factor in fatty acid oxidation, while CPT-1 is a key target gene downstream of PPAR-α. CPT-1 expression in liver is regulated by its upstream factor PPAR-α, and the PPAR-α/CPT-1 pathway in liver lipid metabolism is closely associated with obesity. PPAR-α induces muscle- and liver-specific expression of CPT1 to promote transport of fatty acids to mitochondria and their subsequent β oxidation. PPAR-α can also affect mitochondrial β-oxidation and ω oxidation by regulating acetyl coenzyme A oxidase and cytochrome P450, leading to alteration in lipid metabolism in mitochondria and inhibition of obesity [34]. LPL is a proteolytic enzyme and it is a key enzyme in the lipid metabolism pathway. Its main function is to catalyze decomposition of TG in plasma CM and VLDL into free fatty acids and to promote the transport of proteins, phospholipids, and apolipoproteins. This increases high-density lipoprotein (HDL) levels and inhibits the elevation of TG levels associated with obesity [35]. Decreased LPL activity mitigates degradation and clearance of TG-rich VLDL resulting in increased TG and decreased HDL-C levels in plasma, hyperlipidemia, and obesity [36]. CYP7A1 is a rate-limiting enzyme in bile acid biosynthesis. As a member of liver-specific microsomal cytochrome P450 enzymes, it catalyzes the decomposition of cholesterol to bile acids in liver [37]. Nearly half of the cholesterol in the human body is converted to bile acid under the action of CYP7A1 and is then excreted. Therefore, CYP7A1, a key regulator in the cholesterol synthesis pathway, plays an important role in controlling cholesterol bile acid synthesis and inhibiting development of obesity [38]. In this study, LP-CQPC02 upregulated mRNA and protein expression of LPL, PPAR-α, CYP7A1, and CPT1, and downregulated PPAR-γ and C/EBP-α expression in liver. These reactions alleviated the high-fat diet-induced obesity. Probiotics can significantly reduce the amount of liver enzymes and total cholesterol in obese people. Probiotics can control the accumulation of fat in the liver from the source, so as to play a role in regulating obesity-related expression in the liver, thereby exerting the weight-loss effect of probiotics on the body (Figure 6) [39]. LP-CQPC02 may also regulate obesity-related expression and exert its weight-loss effect through this effect in mice.

## 5. Conclusions

In summary, we found that LP-CQPC02 reduced high-fat diet-induced obesity in mice. LP-CQPC02 also improved abnormal lipid metabolism in the serum and liver and attenuated the liver tissue damage associated with obesity. These results suggest that LP-CQPC02 may be an effective probiotic with the health benefits of preventing dyslipidemia and protecting the liver from high-fat diet/obesity-induced damage. This study could serve as the foundation for further research on LP-CQPC02, but the beneficial effects have only been documented in mice. Additional research and future clinical trials would be needed to verify its applicability in humans.

## Figures and Tables

**Table 1:** Diet formula of mice in this study.

Diet Ingredients (%)	Corn Flour	Bran	Wheatmeal	Soybean Meal	Sucrose	Lard	Premix
10% fat diet	24.89	15.00	7.00	18.50	16.38	1.02	17.21
45% fat diet	7.79	27.00	/	22.00	20.00	19.50	23.71

Premix: trace mineral elements, vitamins, synthetic amino acids.

**Table 2:** Sequences of primers of peroxisome proliferator-activated receptor-alpha (PPAR-α), peroxisome proliferator-activated receptor-gamma (PPAR-γ), cholesterol 7 alpha-hydroxylase (CYP7A1), carnitine palmitoyltransferase 1 (CPT1), CCAAT enhancer-binding protein alpha (C/EBPα), glyceraldehyde-3-phosphate dehydrogenase (GAPDH).

Gene Name	Sequence
*PPAR-α*	Forward: 5′-CCTCAGGGTACCACTACGGAGT-3′
	Reverse: 5′-GCCGAATAGTTCGCCGAA-3′
*PPAR-γ*	Forward: 5′-AGGCCGAGAAGGAGAAGCTGTTG-3′
	Reverse: 5′-TGGCCACCTCTTTGCTGTGCTC-3′
*CYP7A1*	Forward: 5′-AGCAACTAAACAACCTGCCAGTACTA-3′
	Reverse: 5′-GTCCGGATATTCAAGGATGCA-3′
*CPT1*	Forward: 5′‑AAAGATCAATCGGACCCTAGACA-3′
	Reverse: 5′‑CAGCGAGTAGCGCATAGTCA-3′
*LPL*	Forward: 5′‑AGGGCTCTGCCTGAGTTGTA-3′
	Reverse: 5′‑AGAAATCTCGAAGGCCTGGT-3′
*C/EBPα*	Forward: 5′-TGGACAAGAACAGCAACGAGTAC-3′
	Reverse: 5′-GCAGTTGCCCATGGCCTTGAC-3′
*GAPDH*	Forward: 5′-ACCCAGAAGACTGTGGATGG-3′
	Reverse: 5′-ACACATTGGGGGTAGGAACA-3′

**Table 3:** Resistance of lactic acid bacteria to artificial gastric juice and bile salt.

Stain	Tolerance Test of Artificial Gastric Juice (%)	Resistance of *Lactobacillus* to 0.3% Bile Salt (%)
LDSB	37.69 ± 4.52	7.95 ± 0.23
LP-CQPC02	91.88 ± 6.27	18.02 ± 0.26

Values presented are the mean ± standard deviation (*n* = 10/group). LDSB: *Lactobacillus delbruechii* subsp. *bulgaricus*; LP-CQPC02: *Lactobacillus plantarum* CQPC02.

**Table 4:** Organ index of mice in each group (*n* = 10).

Group	Liver Index	Epididymal Fat Index	Perirenal Fat Index
Normal	2.98 ± 0.04 ^e^	1.04 ± 0.04 ^e^	0.13 ± 0.03 ^e^
Model	4.63 ± 0.08 ^a^	2.64 ± 0.11 ^a^	1.39 ± 0.11 ^a^
LP-CQPC02	3.48 ± 0.06 ^d^	1.40 ± 0.10 ^d^	0.32 ± 0.06 ^d^
l-carnitine	3.78 ± 0.07 ^c^	1.64 ± 0.11 ^c^	0.57 ± 0.05 ^c^
LDSB	4.04 ± 0.08 ^b^	2.12 ± 0.14 ^b^	0.93 ± 0.09 ^b^

Values presented are the mean ± standard deviation (*n* = 10/group). ^a–e^ Mean values with different letters in the same column are significantly different (*p* < 0.05) according to Duncan’s multiple range test. LDSB: Mice treated with 1.0 × 10^9^ CFU/kg of *Lactobacillus delbruechii* subsp. *bulgaricus*; LP-CQPC02: Mice treated with 1.0 × 10^9^ CFU/kg of *Lactobacillus plantarum* CQPC02; l-carnitine: Mice treated with 200 mg/kg of l-carnitine.

**Table 5:** Levels of aminotransferase (AST), aminotransferase (AST), high density lipoprotein cholesterol (HDL-C), low density lipoprotein cholesterol (LDL-C), total cholesterol (TC) and triglyceride (TG) in serum of mouse (*n* = 10).

Group	ALT (U/L)	AST (U/L)	HDL-C (mmol/L)	LDL-C (mmol/L)	TC (mmol/L)	TG (mmol/L)
Normal	15.36 ± 1.20 ^e^	12.60 ± 0.77 ^e^	1.19 ± 0.20 ^a^	0.39 ± 0.07 ^e^	1.48 ± 0.25 ^e^	0.40 ± 0.05 ^e^
Model	61.36 ± 2.93 ^a^	49.36 ± 2.02 ^a^	0.29 ± 0.03 ^e^	2.12 ± 0.29 ^a^	5.69 ± 0.61 ^a^	1.84 ± 0.12 ^a^
LP-CQPC02	31.02 ± 2.21 ^d^	20.36 ± 0.83 ^d^	0.49 ± 0.05 ^b^	0.75 ± 0.10 ^d^	2.35 ± 0.26 ^d^	0.65 ± 0.06 ^d^
l-carnitine	38.54 ± 1.82 ^c^	26.39 ± 1.22 ^c^	0.68 ± 0.04 ^c^	1.02 ± 0.09 ^c^	3.11 ± 0.27 ^c^	0.88 ± 0.08 ^c^
LDSB	46.36 ± 1.88 ^b^	36.21 ± 1.73 ^b^	0.86 ± 0.05 ^d^	1.56 ± 0.18 ^b^	4.33 ± 0.20 ^b^	1.25 ± 0.11 ^b^

Values presented are the mean ± standard deviation (*n* = 10/group). ^a–e^ Mean values with different letters in the same column are significantly different (*p* < 0.05) according to Duncan’s multiple range test. LDSB: Mice treated with 1.0 × 10^9^ CFU/kg of *Lactobacillus delbruechii* subsp. *bulgaricus*; LP-CQPC02: Mice treated with 1.0 × 10^9^ CFU/kg of *Lactobacillus plantarum* CQPC02; l-carnitine: Mice treated with 200 mg/kg of l-carnitine.

**Table 6:** Levels of ALT, AST, HDL-C, LDL-C, TC and TG in hepatic tissue of mouse (*n* = 10).

Group	ALT (U/gprot)	AST (U/gprot)	HDL-C (U/gprot)	LDL-C (mmol/gprot)	TC (mmol/gprot)	TG (mmol/gprot)
Normal	2.36 ± 0.19 ^e^	3.02 ± 0.36 ^e^	1.29 ± 0.22 ^a^	1.48 ± 0.25 ^e^	28.33 ± 3.68 ^e^	2.56 ± 0.31 ^e^
Model	12.38 ± 1.52 ^a^	9.36 ± 0.61 ^a^	0.52 ± 0.09 ^e^	3.69 ± 0.31 ^a^	91.05 ± 5.22 ^a^	11.86 ± 0.74 ^a^
LP-CQPC02	3.45 ± 0.24 ^d^	4.25 ± 0.56 ^d^	0.79 ± 0.05 ^d^	2.02 ± 0.38 ^d^	40.14 ± 2.15 ^d^	4.15 ± 0.32 ^d^
l-carnitine	4.12 ± 0.38 ^c^	6.32 ± 0.48 ^c^	0.91 ± 0.05 ^c^	2.71 ± 0.19 ^c^	48.92 ± 3.11 ^c^	6.17 ± 0.49 ^c^
LDSB	7.39 ± 0.62 ^b^	8.02 ± 0.41 ^b^	1.11 ± 0.04 ^b^	3.15 ± 0.22 ^b^	66.21 ± 3.58 ^b^	9.30 ± 0.55 ^b^

Values presented are the mean ± standard deviation (*n* = 10/group). ^a–e^ Mean values with different letters in the same column are significantly different (*p* < 0.05) according to Duncan’s multiple range test. LDSB: Mice treated with 1.0 × 10^9^ CFU/kg of *Lactobacillus delbruechii* subsp. *bulgaricus*; LP-CQPC02: Mice treated with 1.0 × 10^9^ CFU/kg of *Lactobacillus plantarum* CQPC02; l-carnitine: Mice treated with 200 mg/kg of l-carnitine.

**Table 7:** Cytokine levels of tumor necrosis factor alpha (TNF-α), interferon gamma (IFN-γ), interleukin 1 beta (IL-1β), interleukin 4 (IL-4), interleukin 6 (IL-6) and interleukin 10 (IL-10) in serum of mouse (*n* = 10).

Group	TNF-α (pg/mL)	IFN-γ (pg/mL)	IL-1β (pg/mL)	IL-4 (pg/mL)	IL-6 (pg/mL)	IL-10 (pg/mL)
Normal	52.20 ± 2.52 ^e^	43.85 ± 1.92 ^e^	22.69 ± 0.62 ^e^	58.36 ± 1.59 ^a^	42.61 ± 2.28 ^e^	118.61 ± 9.21 ^a^
Model	97.86 ± 3.62 ^a^	106.36 ± 5.23 ^a^	79.69 ± 3.20 ^a^	22.56 ± 0.36 ^e^	86.39 ± 3.38 ^a^	40.88 ± 2.12 ^e^
LP-CQPC02	66.58 ± 2.06 ^d^	55.15 ± 3.23 ^d^	46.32 ± 3.28 ^d^	50.33 ± 2.17 ^b^	51.88 ± 2.82 ^d^	92.06 ± 4.12 ^b^
l-carnitine	75.36 ± 2.13 ^c^	69.18 ± 4.02 ^c^	57.89 ± 2.93 ^c^	42.01 ± 2.33 ^c^	63.84 ± 3.01 ^c^	76.52 ± 3.91 ^c^
LDSB	84.56 ± 2.40 ^b^	81.09 ± 3.54 ^b^	64.18 ± 2.67 ^b^	32.51 ± 2.69 ^d^	74.19 ± 2.63 ^b^	60.85 ± 4.12 ^d^

Values presented are the mean ± standard deviation (*n* = 10/group). ^a–e^ Mean values with different letters in the same column are significantly different (*p* < 0.05) according to Duncan’s multiple range test. LDSB: Mice treated with 1.0 × 10^9^ CFU/kg of *Lactobacillus delbruechii* subsp. *bulgaricus*; LP-CQPC02: Mice treated with 1.0 × 10^9^ CFU/kg of *Lactobacillus plantarum* CQPC02; l-carnitine: Mice treated with 200 mg/kg of l-carnitine.
